# Development and psychometric testing of the Knowledge, Attitudes and Practices (KAP) questionnaire among student Tuberculosis (TB) Patients (STBP-KAPQ) in China

**DOI:** 10.1186/s12879-018-3122-9

**Published:** 2018-05-08

**Authors:** Yahui Fan, Shaoru Zhang, Yan Li, Yuelu Li, Tianhua Zhang, Weiping Liu, Hualin Jiang

**Affiliations:** 1 Department of Nursing, Xi’an Jiaotong University, Xi’an, 710061 China; 2 Shaanxi Provincial Institute for Tuberculosis Control and Prevention, Xi’an, 710048 China

**Keywords:** Student, Tuberculosis (TB) patients, Knowledge, attitudes and practices (KAP), Questionnaire development, Psychometric testing

## Abstract

**Background:**

TB outbreaking in schools is extremely complex, and presents a major challenge for public health. Understanding the knowledge, attitudes and practices among student TB patients in such settings is fundamental when it comes to decreasing future TB cases. The objective of this study was to develop a Knowledge, Attitudes and Practices Questionnaire among Student Tuberculosis Patients (STBP-KAPQ), and evaluate its psychometric properties.

**Methods:**

This study was conducted in three stages: item construction, pilot testing in 10 student TB patients and psychometric testing, including reliability and validity. The item pool for the questionnaire was compiled from literature review and early individual interviews. The questionnaire items were evaluated by the Delphi method based on 12 experts. Reliability and validity were assessed using student TB patients (*n* = 416) and healthy students (*n* = 208). Reliability was examined with internal consistency reliability and test-retest reliability. Content validity was calculated by content validity index (CVI); Construct validity was examined using exploratory factor analysis (EFA) and confirmatory factor analysis (CFA); The Public Tuberculosis Knowledge, Attitudes and Practices Questionnaire (PTB-KAPQ) was applied to evaluate criterion validity; As concerning discriminant validity, T-test was performed.

**Results:**

The final STBP-KAPQ consisted of three dimensions and 25 items. Cronbach’s α coefficient and intraclass correlation coefficient (ICC) was 0.817 and 0.765, respectively. Content validity index (CVI) was 0.962. Seven common factors were extracted by principal factor analysis and varimax rotation, with a cumulative contribution of 66.253%. The resulting CFA model of the STBP-KAPQ exhibited an appropriate model fit (χ2/df = 1.74, RMSEA = 0.082, CFI = 0.923, NNFI = 0.962). STBP-KAPQ and PTB-KAPQ had a strong correlation in the knowledge part, and the correlation coefficient was 0.606 (*p* < 0.05). Discriminant validity was supported through a significant difference between student TB patients and healthy students across all domains (*p* < 0.05).

**Conclusions:**

An instrument, “Knowledge, Attitudes and Practices Questionnaire among Student Tuberculosis Patients (STBP-KAPQ)” was developed. Psychometric testing indicated that it had adequate validity and reliability for use in KAP researches with student TB patients in China. The new tool might help public health researchers evaluate the level of KAP in student TB patients, and it could also be used to examine the effects of TB health education.

## Background

Since 1970, Knowledge, attitudes and practices (KAP) research has been the primary educational intervention strategy for tuberculosis (TB) control worldwide [1]. Several studies had shown that, the level of KAP in individuals was linked to efficient management of illness, response to medical treatment, and promotion of one’s own health [2–5]. Lower KAP level had been one of the main indicators of poor health, inefficient health care use, the decrease of the disease screening rate, and maladaptive disease preventive behavior [6–8].

Tuberculosis (TB) has existed for millennia and remains a major global health problem. It caused ill-health in millions of people each year and was one of the top 10 causes of death worldwide, ranking above HIV/AIDS as one of the leading causes of death from an infectious disease [9]. Globally in 2015, there was an estimated 10.4 million incident cases of TB, of these, China, India and Indonesia alone accounted for 45% [9]. In Shaanxi Province of China, from 2011 to 2015, the number of student TB patients was always higher than 1300 annually, accounted for 6.17-6.78% of total TB cases [10, 11]. At the same time, TB transmission was ongoing in schools. A total of 44 student TB patients were found and diagnosed during TB outbreak in a middle school in Jinan, Shandong Province from April 2013 to November 2014 [12]. A military academy in Guangdong Province cumulatively reported 44 cases of TB from January to July in 2014 [13]. From February to April in 2015, 11 student TB patients were found in a middle school in Liaoyang, Liaoning Province [14]. Even in many lower TB burden countries, such as Britain and the United States, epidemic of student TB has also been reported [15, 16].

TB outbreaking in schools is extremely complex, and presents a major challenge for global public health, and the outbreak highlights the need for innovative TB prevention and control strategies in such settings. As most students are from a high risk population group, they need some prevention strategies including prompt referral of symptomatic cases to TB services, and to be raised awareness about TB. The survey of Ridzon R showed that students who have significantly higher knowledge scores were more likely to find TB timely [17]. Donald et al. [18] pointed out that TB education among teenagers could help patients successfully complete the TB treatment. Chen et al. [19] reported that students had delayed diagnosis due to the lack of TB prevention knowledge. All of these suggest that it is necessary to evaluate the level of KAP in student TB patients, which can help us carry out the targeted health education and prevent future outbreaks.

### Overview of existing measurement instruments

There are many ways to assess the level of KAP in student TB patients, and self-administration of questionnaire is the most common approach. Iranish scholar Fatemah et al. [20] and Turkish scholar Semiha et al. [21] investigated the KAP level in student TB patients using a questionnaire with a description of composition contents only; Marguerite et al. [22] conducted a cross-sectional survey about TB KAP in health professional students throughout California area, and the questionnaire was only assessed with the clarity and face validity; Roman scholar Mushta et al. [23] carried out a survey of TB KAP level in medical students and the general population, respectively, a pilot test was only conducted for their questionnaires, and the results had not been reported; Daniel et al. [24] investigated the TB KAP level of a community in Ethiopia, Somalia, but the reliability and validity of the questionnaire had not been estimated.

In China, the questionnaire used most widely is the public TB KAP questionnaire (PTB-KAPQ) [25]. Qingdao University has developed a TB KAP questionnaire for university students in 2012 [26]. However, these instruments are generic, none of them were specifically designed for assessing student TB patients’ KAP level. In terms of TB knowledge, student TB patients should understand basic symptoms of TB, treatment process, the correct handling of the side effects of drugs, the free treatment policy and principles of chemical treatment of TB (early, joint, right-amount, regular, whole-journey). Regard to TB attitudes, they should learn that hiding their disease is not correct, and it is necessary to remind close contacts of an inspection. Behavior is especially important, we need to know whether student TB patients have the high-risk behaviors in TB spread, such as not wearing masks in public, coughing and sneezing in front of others.

This study was aimed at developing Knowledge, Attitudes and Practices (KAP) Questionnaire among Student Tuberculosis (TB) Patients (STBP-KAPQ), and evaluated its psychometric properties. The questionnaire will help TB medical professional identify not only student TB patients with poor level of KAP, but also help them to design and implement targeted interventions to improve the level of KAP.

### Framework for the development of STBP-KAPQ

“KAP theory” is a health behavior change theory, proposed by western scholars in the 1960s [27], in which the changes of human behavior are divided into three successive processes: the acquisition of knowledge, the generation of attitudes and the formation of behavior. The theory presents the progressive relationship among knowledge, attitudes and behavior as follows: knowledge is the foundation of behavior change, and belief and attitudes are the driving force of behavior change. “Health belief model” was put forward in the 1950s [28], which pointed out that the formation of health belief played a key role for people to accept the persuasion, change the bad behavior, and adopt the healthy behavior. Therefore, the “KAP theory” and “Health belief model” were adopted to guide the development of the STBP-KAPQ.

## Methods

### Questionnaire development procedures

#### Phase 1: Item construction

##### A. Item pool's three sources:

1. WHO “A guide to develop KAP surveys” (World Health Organization 2008) [29]; Collecting as many terms as possible that were considered essential knowledge for student TB patients by referring to books on health, health-related magazines, and leaflets distributed by hospitals and government institutes, such as “China TB prevention and control work guide (2008 edition)”, “school TB prevention and control work manual”.
2. A review of published research on KAP definitions and concepts, and on its measurement.
3. Early interview of the research team:

Following the principle of informed consent, confidentiality and voluntary, the research team once performed an individual in-depth interview with 17 college students in 2013 and 22 high school students in 2015 after the TB epidemic emerged. The interview topics included general information, the medical behavior, medication compliance, knowledge about the TB control, the difficulties during treatment, the psychological burden after TB diagnosis and so on.

##### B. Two-round expert consultation

*Participants*

According to the inclusion criteria: (a) engaging in the TB prevention and control, clinical diagnosis and treatment, health education and school TB control management; (b) working for more than 10 years; (c) the subtropical high titles and above. In the Delphi method, 12 experts (4 TB prevention and control experts, 1 public health professionals, 2 clinical doctors, 2 health education and health promotion experts, 3 school TB prevention and control of management specialists), were recruited. They were from the Ministry of Health, the Chinese Center For Disease Control and Prevention (CDC), the Provincial CDC and the schools.

*Delphi round 1*

Round 1 of the Delphi sent a copy of the 23-items questionnaire to the expert panel (*n* = 12) by e-mail. Experts voted on a five-point scale (0 to 4, where 0 = very unimportant and 4 = very important) showing the extent to which they thought each of the 23 questions should be included in the S-TBKAPQ. Experts were asked to return within two weeks, and a reminder e-mail was sent if no response had been made after one week.

*Delphi round 2*

In Round 2 of the Delphi, responses of round 1 were collated and the questionnaire was amended as appropriate, then a amended questionnaire was sent to the same expert panel (*n* = 12) via e-mail. Experts were asked to re-rate the questions on the same five-point scale in light of the results and comments from Round 1. Experts were still asked to return within two weeks, and a reminder e-mail was also sent if there was no response after one week.

*Evaluation index*

- Positive coefficient: the positive coefficient refers to the extent of experts’ concern about the research, evaluated by the recovery rate of the questionnaire. It is generally believed that the recovery rate of 50% is the lowest proportion can be analyzed, 60% is a good result, and 70% is a rather good result [30].
- The authoritative coefficient: the authoritative coefficient (Cr) was generally decided by two factors: the judgment criterion (Ca) and the familiarity (Cs). The calculation formula of authoritative coefficient was as follows: Cr = (Ca + Cs) /2, the greater the Cr, the greater the degree of authority [31].

##### C. The screening of items

After each round of expert consultation, the importance of expert’s judgment to items was computed. According to a five-point scale (0 to 4, where 0 = completely unimportant and 4 = completely important), experts were required to give the corresponding score in terms of the importance of items, and then the items were screened according to the central tendency and dispersion degree.

The central tendency was evaluated by the item selection rate (The item selection rate = the number of experts who rated items with a score of 3 (important) or 4 (very important) /the total number of experts× 100%). If the item selection rate was < 80%, it would be deleted [32]. The discrete degree was measured using the coefficient of variation (the ratio of standard deviation and arithmetic average of the item importance score). If the variable coefficient was ≥0.2, it would be deleted [32].

The initial draft of the questionnaire was produced through above procedures.

#### Phase 2: Pilot study

A similar sample of 10 student TB patients from Xi’an Chest Hospital enrolled in the pilot study, including two junior high school students, four senior high school students and four college students. They were asked to complete the initial draft of the questionnaire, and afterwards they were asked to provide their comments about problems in completing it, including whether it was clear and understandable, and also whether the content was complete and relevant. After these producers, the final version of the instrument to test validity and reliability was created.

#### Phase 3: The test of validity and reliability

##### *Participants*

The sample size recommended is 5 to 10 participants per item. As the number of the items was intended to be 25, a sample size estimated was 125 to 250 [33]. It is widely acknowledged that at least 100 samples are required, in order to establish an accurate inference in exploratory factor analysis (EFA) [34]. In addition, in order to evaluate confirmatory factor analysis (CFA), a minimum sample size of 200 is needed to gain reliable results [35]. Considering a 20% non-response rate, the minimum sample size was set at 390 participants.

According to the inclusion and exclusion criteria: (a) who were middle school and college students; (b) with a confirmed TB; (c) who was reported in the TB registry system; (d) whose treatment was undergoing or complete; and (e) who was voluntary to participate in the study; (f) patients who suffered from other serious diseases or had cognitive impairment were excluded from the study. 416 student TB patients by convenience sample (including 64 junior high school, 129 high school students and 223 college students) were recruited, which were randomly divided into two parts (N1 = 206, N2 = 210), the former for exploratory factor analysis (EFA) and the latter was used in the confirmatory factor analysis(CFA), respectively. To distinguish clearly between two different types about measuring indexes, the sample size of two groups recommended are equal [36], so 208 healthy students were recruited as a comparison group to assess the discriminant validity, matched by sex and grade, in similar proportions as the 206 student TB patients of the exploratory factor analysis (EFA). It’s requested that the sample size in the test-retest reliability is not fewer than one over ten of the total research objects, and the larger the sample size the better [37], so 50 student TB patients were selected for evaluation of the test-retest reliability.

##### *Measures*

The aspects of reliability (internal consistency reliability, re-test reliability,) and validity (content validity, construct validity, criterion validity, discriminant validity) of the questionnaire were tested.

##### *Data collection*

The research team who collected the questionnaires was made up of five trained researchers. Except for the chairman, two members as a group went to the target locations respectively. Written informed content was obtained prior to the survey. We handed out the questionnaires to participants face-to-face and one-to-one, and withdrawed them on the spot. At this stage, explanations were necessary to ensure that the participants understood the purpose and importance of the study. With regard to respondents who had lower reading comprehension, researchers would read the instructions and questions without any additional interpretation or explanation. In order to improve the response rates, all participants received a beautiful notebook or a red packet as reward before the survey. Participants independently completed the questionnaire, and all the questionnaires were anonymous. Questionnaires with missing response ≧ 20% or apparently unreliable responses were considered ineligible and would be removed before analysis, (for instance, the choices in the questionnaire were all the same, with no changes; or the logic of the answer was chaotic, inconsistent and contradictory with itself), in order to ensure the quality of data and the rationality of statistical results.

##### *Data analysis*

Data were analyzed using SPSS18.0 and AMOS 18.0 software. Descriptive statistics was used to outline the demographic characteristics. Cronbach’α coefficient was computed for internal consistency reliability, and intraclass correlation coefficient (ICC) was used to evaluate test-retest reliability. Content validity was measured by content validity index (CVI). Construct validity was examined by exploratory factor analysis (EFA) and confirmatory factor analysis (CFA). For criterion validity of the STBP-KAPQ, a Spearman correlation coefficient was calculated, and T-test was employed for a group comparison between the student TB patients and healthy students, providing an evidence for discriminant validity.

## Results

### Expert characteristics and the result of consultation

The average age of the experts was 49.83 ± 10.85, and the average serving time was 15.25 ± 7.97 years. Experts in title of a senior professional post accounted for 75%. The positive coefficient was 100%, and the authoritative coefficient of this study was 0.866.

### The result of item screening

Five items were added after the first round expert consultation, including the knowledge items associated with TB examination, the consequences of drug withdrawal,the drug adverse reactions, the attitudes item about spreading TB knowledge as a volunteer, and the behavior item of what to do with the sputum. Three attitudes items associated with TB prevention and control were removed. There were 25 items in the second round, which were judged again by the experts, and the initial draft of the questionnaire was produced.

### The result of pilot study

They completed the initial draft questionnaire with no item non-response within 7–15 min. The instrument was considered not long and easy to complete. All items were considered relevant to the aim of study, and no suggestions were made regarding the exclusion of any items or the addition of new items. Fuzzy expression statements of some items were revised, in case of causing unease.

### The result of the STBP-KAPQ

According to the opinions of experts and 10 student TB patients in the pilot study, a total of 25 preliminary items were selected, reflecting four constructs of STBP-KAPQ as follows: general demographic information, TB knowledge, attitudes and behavior related to the TB prevention and control. One point was given if there was a right selection in the knowledge part, and items of the attitudes (two items not scoring) and practices were stated as propositions with a labeled five-point scale (1 = very disagreed, 5 = very agreed) and a four-point scale (1 = never, 4 = always), respectively. Some of the items were reverse scoring. A final version of the questionnaire was created to evaluate validity and reliability.

### The results of validity and reliability

#### Participant characteristics

Within the 416 student TB patients (210 college students, 206 middle school students), 416 questionnaires were distributed to student TB patients, only 408 of the recovered questionnaires were valid, and the recovery rate was 98.72%. Moreover, 67.5% of the student TB patients were males, and more than half of them were college students (Table 1). For 208 healthy students (108 college students, 100 middle school students), 208 questionnaires were distributed and withdrawed, all of them were valid, so the recovery rate was 100%. The healthy students in this study were recruited by matching sex and grade to the group of student TB patients. Chi-squared test, T-test, analyses of variance (ANOVA) and Mann-Whitney U test were conducted to examine the characteristics differences between the student TB patients group and the healthy one. As expected, there were no significant differences observed between the two groups in terms of gender (*p* > 0.05), grade (*p* > 0.05), personal monthly cost (*p* > 0.05), TB contact history (*p* > 0.05) and TB healthy education (*p* > 0.05).

**Table 1:** Demographic characteristics of participants

	Student TB patients	Healthy students	χ^2^/T/Z /F	P
	(*n* = 408)	(*n* = 208)		
Demographic variables	n(%)	n(%)		
Gender			0.569	0.401
Male	275(67.5)	123 (59.1)		
Female	133(32.5)	85 (40.9)		
Education				
Junior high school	72 (17.5)	44 (21.2)	−4.639	−0.201
Senior high school	101 (24.8)	56 (26.9)		
College	235 (57.7)	108 (51.9)		
Family place			2.485	0.115
Urban area	158 (38.8)	109 (52.4)		
Rural area	250 (61.2)	99 (47.6)		
Personal monthly cost			−7.329	−2.431
< 500	214 (52.4)	78 (27.5)		
500-1000	115 (28.2)	86 (41.3)		
1000-1500	61 (15.0)	23 (11.1)		
> 1500	18 (3.4)	21 (20.1)		
TB contact history				
Yes	93 (22.8)	25 (12)	3.314	0.069
No	315 (77.2)	183 (88)		
TB healthy education				
Yes	339 (83.0)	168 (80.8)	2.876	0.090
no	69 (17.0)	40 (19.2)		

#### The result of reliability

The Cronbach’α coefficient was 0.817, ranging from 0.793 to 0.817, and it was slightly changed once one item was deleted from the three sections (knowledge, attitudes and behaviors). To evaluate test–retest reliability, 50 student TB patients were remeasured after 2 weeks, and the intraclass correlation coefficient was 0.765.

#### The result of validity

##### Construct validity

Exploratory factor analysis (EFA) and confirmatory factor analysis (CFA) were used to explore the appropriate construct of STBP-KAPQ.

##### The result of exploratory factor analysis

The Kaiser-Meyer-Olkin (KMO) measure of sampling adequacy, and the Bartlett’s test of sphericity were used prior to factor analysis, to ensure that the data from student TB patients were appropriate for conducting factor analysis. The factor analysis was based on the following criteria: (a) A bigger KMO value: KMO value should be between 0 and 1, the greater its value the better factor analysis results. If KMO value is < 0.5, it is unsuitable for factor analysis. (b) Significant Bartlett ball test (*p* < 0.05), which was used to examine whether the factor was independent. In the present study, the KMO value was 0.687, Bartlett’s test of sphericity was found to be significant (χ^2^ = 81,797.730, *p* < 0.05). Therefore, the exploratory factor analysis could be conducted on this study.

**Fig. 1:**
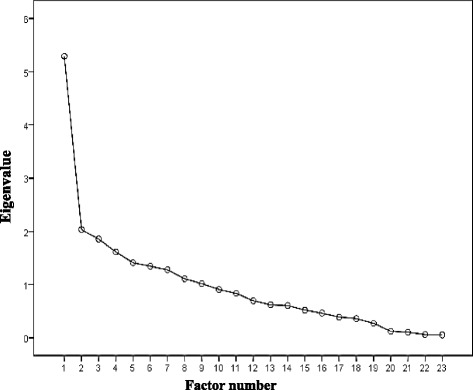
Scree Plot

Principal component analysis with biggest variance orthogonal rotation was applied to determine the underlying factor structure of the 25 items. The factor loading of all questionnaire items were not present cross, and the factor loading values were more than 0.4, ranging from 0.428 to 0.924 (Table 2). Since eigenvalues were more than 1, seven meaningful factors were extracted, which explained 66.253% of the total variance (Table 3). Factor 1 (adverse drug reaction) was related to side effects of drug, and it had the maximum contribution (21.541%). Factor 2 (standard treatment) was associated with TB cure. Factor 3 (active notification) showed the importance of TB notification, and factor 4 (positive behavior) was associated with the positive life behavior of TB. Factor 5 (regular medication) was connected with the entire regular medication, factor 6 (active prevention) was associated with active prevention of TB, and factor 7 (core knowledge) was about the TB understanding. The result of scree plot also illustrated that the seven factors should be extracted (Fig. 1).

**Table 2:** Factor loading results after rotating

Item		Factors						
		F1	F2	F3	F4	F5	F6	F7
Factor 1(F1): adverse drug reaction(3 items)								
K6	Common adverse reactions of anti-TB drugs	.858						
K7	What should be done due to adverse reaction of drugs	.851						
P8	Will you reduce or stop taking the medicine when feeling unwell	.831						
Factor 2(F2): standard medication(4 items)								
K10	the most critical measure of curing TB		.844					
K5	How long will TB patients need to take the medicine to heal		.822					
K8	the consequences of self-stopping taking the medicine		.705					
K9	Whether can TB be cured		.493					
Factor 3(F3): active notification (2 items)								
A4	You should remind people of TB relevant checks who are in close contact with you			.924				
A3	You should tell counselor that you are suffering from TB			.787				
Factor 4(F4): positive behavior (5 items)								
P2	Will you wear a mask when going to public during sickness				.905			
P5	How do you handle the sputum during sickness				.802			
P3	Will you cover mouth and nose when speaking,coughing, sneezing during sickness				.786			
P10	Which have you done according to the recovery behaviors				.744			
P4	Would you go to the hospital to review regularly				.711			
Factor 5(F5): regular medication (3 items)								
P7	Have you ever missed a drug during the treatment					.799		
P6	Would you need someone to supervise your medication					−.675		
P9	Will you stop taking the medicine yourself if the condition is controlled but not at a full course of treatment					−.568		
Factor 6(F6): active prevention (2 items)								
P1	Have you actively learned about TB						.806	
A5	Are you willing to be a TB volunteer to promote TB knowledge						.454	
Factor 7(F7): core knowledge (4 items)								
K4	The accessory examinations of TB							.690
K3	The main symptoms of TB							.589
K2	Students who are susceptible to TB							.551
K1	The primary transmission route of TB							.428

**Table 3:** Factor characteristic value and variance contribution rate

Factors	Characteristics	Variance contribution rate (%)	Cumulative contribution rate (%)
F1	4.954	21.541	21.541
F2	2.327	10.116	31.657
F3	2.043	8.883	40.540
F4	1.594	6.930	47.470
F5	1.588	6.904	54.374
F6	1.406	6.114	60.488
F7	1.326	5.765	66.253

##### The result of confirmatory factor analysis

Confirmatory factor analysis was used to establish the most appropriate factor structure of the STBP-KAPQ, model fit was considered acceptable if χ^2^/df < 2, comparative fit index (CFI) > 0.9, root mean square error of approximation (RMSEA) < 0.06 [38–40]. The 7-factor model was found to fit the data much better with a smaller model χ^2^/df statistics (χ2/df = 1.74) and stronger model fit indexes (CFI = 0.923, RMSEA = 0.082), than the original model (CFI = 0.802). As expected, the STBP-KAPQ with 7-factor structure model was an acceptable fit model.

##### Content validity

Content validity reflects the representativeness of the questionnaire items. The higher the content validity index is, the better item’s representativeness is. In this study, the content validity index was calculated by the proportion of the items with a rating of 3 or 4 by all experts. After evaluated by above-mentioned 12 experts, the content validity index was 0.962, which achieved the criterion for content validity, showing that the questionnaire items were accurate and comprehensive, and it covered all TB-related knowledge, attitudes and behaviors that the student TB patients should learn.

##### Criterion validity

To test criterion validity of the STBP-KAPQ, The Public Tuberculosis Knowledge, Attitudes and Practices Questionnaire (PTB-KAPQ) was used as a calibration standard questionnaire. Through Spearman analysis, the correlation coefficient between STBP-KAPQ and the PTB-KAPQ was 0.464 (*p* < 0.05), and there was a strongest correlation between the knowledge section and the PTB-KAPQ questionnaire (*r* = 0.606, *p* < 0.01). Moreover, there was a weaker correlation between attitudes, behavior sections and the PTB-KAPQ (Table 4).

**Table 4:** Correlation between STBP-KAPQ and PTB-KAPQ

	PTB-KAPQ
**STBP-KAPQ**	0.464*
Knowledge	0.606**
Attitudes	0.293*
Practices	0.307*

*p < 0.05; **p < 0.01

##### Discriminant validity

For student TB patients, the mean (SD) scores for knowledge, attitudes and practices were 14.49 (1.850), 12.06 (1.376) and 20.40 (4.282), respectively. Healthy students indicated a lower mean (SD) score, which were 13.31 (1.740), 11.51 (1.630), 19.28 (4.957), respectively. There was a significant difference between the two groups (*p* < 0.05) (Table 5).

**Table 5:** Difference between student TB patients and healthy students

	Student TB patients		Healthy students		T	P
	Mean	SD	Mean	SD		
Knowledge	14.49	1.850	13.31	1.740	6.646	< 0.001**
Attitudes	12.06	1.376	11.51	1.630	3.699	< 0.001**
Practices	20.40	4.282	19.28	4.957	2.447	0.015*

*p < 0.05; **p < 0.01

## Discussion

In this study, we produced a 25-item STBP-KAPQ and showed that STBP-KAPQ was fairly consistent, reliable and valid. The STBP-KAPQ is the first measurement fitting situations of student TB patients in China.

### The evaluation of reliability

Cronbach’α coefficient reflects the internal consistency of questionnaire. In general, Cronbach’α coefficient between 0.65-0.70 is the minimum acceptable value, 0.70-0.80 is rather good, and 0.80-0.90 is the best. Our results showed that the Cronbach’α coefficient of the questionnaire was 0.817, revealing that STBP-KAPQ had a good internal consistency reliability. For a new development of the measurement tool, it is believed that the retest reliability need to be more than 0.7. The retest correlation coefficient of our study was 0.765, revealing that the retest reliability of questionnaire was up to the requirements of psychological measurement, showing a good stability.

### The evaluation of validity

Our results showed that the content validity index was 0.927, consistent with the requirement of a content validity index of at least 0.8, suggesting that the items could well reflect the KAP condition in student TB patients. In general, if the cumulative contribution rate above 40%, and each item on the corresponding factor owns enough loading (> 0.4), the factor is acceptable and the relationship between the item and factor is meaningful [41]. Our study adopted the principal component factor analysis method, and seven common factors were extracted. The cumulative contribution rate was 66.253%, and the corresponding factor loading of each item was > 0.4, revealing that each item in the common factor distribution conformed to the theoretical construction of questionnaire. In confirmatory factor analysis, the χ2/df was 1.74, the comparative fit index (CFI) was 0.923, and the root mean square error of approximation (RMSEA) was 0.082. The fitting indexes of the model reached significant level, therefore, the modified 7-factor model was the final model. The results of confirmatory factor analysis and exploratory factor analysis displayed that the questionnaire had reasonable construct validity.

In the present study, we used the Public Tuberculosis Knowledge, Attitudes and Practices Questionnaire (PTB-KAPQ) as the criterion of STBP-KAPQ. Based on the analysis of criterion validity, there was a correlation between two questionnaires (*r* = 0.464, *p* < 0.05). The section of knowledge was highly related with the public questionnaire (*r* = 0.606, *p* < 0.01), but the sections of attitudes and practices exhibited a weaker correlation with the public questionnaire. This finding was consistent with our prediction. Our questionnaire aimed to develop for the student TB patients, leading to some different emphasis on the specificity of KAP level. For example, in the section of attitudes, we tended to evaluate whether student TB patients were willing to tell the teacher the situation of their sickness, to have an inspection, and to initiatively expand TB knowledge. In the behavior section, considering that the treatment of anti-TB needs at least 6 months [42], and there are more side effects, we added some items in our questionnaire, such as whether they would countercheck on time; decreased the times of drug or stopped taking medicine on their own. These contents were not covered in the PTB-KAPQ. Therefore, although this questionnaire was consistent with the PTB-KAPQ, they were not exactly the same. This prompted the specificity and uniqueness of STBP-KAPQ, and it also reflected the necessity to develop this questionnaire.

To evaluate the discriminant validity of STBP-KAPQ, we examined the discriminability of STBP-KAPQ in student TB patients and healthy students. Our results revealed that significant difference existed between the two groups on the sections of KAP. In terms of knowledge level, as expected, the scores of healthy students were significantly lower than those of student TB patients (*p* < 0.05), which was consistent with the 2006 public TB KAP survey conducted by Chinese CDC [43]. Here is the probable reason that students are generally more active to learn TB prevention and control knowledge after sick, more eager to figure out what TB is, while healthy students think TB is far away from themselves, so they don’t have a high enthusiasm to understand TB. In the attitudes and practices sections, the scores of students TB patients were higher than those of healthy students (*p* < 0.05). The reason may be student TB patients have set up the healthy attitudes and behavior in the standardized treatment, and they have learnt about how to promote the recovery of the disease. However, healthy students don’t have relevant consciousness about these without an experience of attack by TB, which were consistent with the survey results of Yang Yunbin [44].

## Conclusion

An instrument, which may be useful as an easy-to-use self-report measure of student TB patients’ KAP towards TB, was developed and tested in our study. The analyses of reliability and validity demonstrated the strong psychometric properties of the STBP-KAPQ. The STBP-KAPQ supplements the limitations of current available TB KAP measures. Public health professionals can use the STBP-KAPQ to identify KAP level among student TB patients. The data may be used as the basis to improve TB prevention and control.

### Limitations

Student TB patients were recruited using convenience sampling from the registry system of Shaanxi Provincial Institute for Tuberculosis Control and Prevention, and it was likely that patients not in the system were excluded, whose KAP was more scarce. The sample collection of this study only covered Shaanxi Province, taking this into consideration, future studies of this instrument will have to ensure the representativeness of study samples with diverse provinces and regions.

## Abbreviations

CDC: Chinese Center For Disease Control and Prevention
CFA: Confirmatory factor analysis
CFI: Comparative fit index
CVI: Content validity index
EFA: Exploratory factor analysis
ICC: Intraclass correlation coefficient
KAP: Knowledge, attitudes and practices
KMO: Kaiser-Meyer-Olkin
NNFI: non-normed fit index
RMSEA: Root-Meta-Square Error of Approximation
TB: Tuberculosis
WHO: World Health Organization
